# Case Report: Severe sepsis due to imported scrub typhus in a German traveler returning from Vietnam

**DOI:** 10.3389/fmed.2026.1772790

**Published:** 2026-05-07

**Authors:** Luis Hinterwaldner, C. Corredor Obregon, Sabine Zange, Eva Ortner, Fabian Kleinhenz, Laura Wagner, C. D. Spinner, Ulrich Mayr, Tobias Lahmer, Jochen Schneider, Johanna Erber

**Affiliations:** 1 TUM School of Medicine and Health, Clinical Department of Internal Medicine II, TUM University Hospital, Munich, Germany; 2 TUM School of Medicine and Health, Institute of Medical Microbiology, Immunology and Hygiene, TUM University Hospital, Munich, Germany; 3 Bundeswehr Institute of Microbiology, Munich, Germany

**Keywords:** case report, imported infection, *Orientia tsutsugamushi*, scrub typhus, sepsis

## Abstract

Scrub typhus, caused by the obligate intracellular bacterium *Orientia (O.) tsutsugamushi*, is an under-recognized cause of febrile illness in travelers returning from endemic regions of the Asia–Pacific. We report the case of a 39-year-old woman who presented with high-grade fever, headache, and hepatitis with coagulopathy following travel to Vietnam. Her condition deteriorated rapidly progressing to sepsis. Laboratory investigations revealed hemolysis with mild anemia, thrombocytopenia, as well as markedly elevated liver enzymes and inflammatory markers. Computed tomography demonstrated extensive bilateral pulmonary infiltrates and pleural effusions consistent with a clinically impending acute respiratory distress syndrome (ARDS). A positive polymerase chain reaction (PCR) for *O. tsutsugamushi* from EDTA blood confirmed the diagnosis. The patient improved under the empirically initiated doxycycline therapy. This case highlights the importance of considering scrub typhus as a potentially life-threatening yet treatable cause of febrile illness in travelers returning from endemic regions. Given its diverse clinical manifestations and lack of coverage by beta-lactams that are usually applied for empirical sepsis therapy, heightened clinical awareness and timely diagnostic testing are crucial – particularly in the context of expanding endemic areas and increasing global mobility.

## Introduction

Scrub typhus, also known as Tsutsugamushi disease, is a mite-borne zoonosis caused by the obligate intracellular, gram-negative parasite *Orientia tsutsugamushi*. It is endemic throughout the Asia-Pacific “Tsutsugamushi triangle,” which extends from northern Japan and far-eastern Russia to northern Australia and Pakistan (1). A rising trend has been noted in recent years, with newly recognized areas of transmission emerging in regions such as the United Arab Emirates, Chile, and Kenya (2–4). It is estimated that over one billion people reside in endemic regions worldwide, with roughly one million infections and 150.000 deaths occurring annually, although confirmed numbers are far lower due to underreporting and limited diagnostic validation (5, 6). A wide phylogenetic range of *Orientia tsutsugamushi* variants has been observed in several molecular-epidemiological investigations in Vietnam as well as the endemic region in general, with predominant and stable strains ‘Karp’, ‘Kato’ and ‘Gilliam’, thus used as prototype strains for detection (7–9). Transmission occurs through the bite of the larval stage (“chigger”) of trombiculid mites, which act both as vector and reservoir. The infection typically follows exposure to grassy or forested environments, often during outdoor recreational activities or agricultural work (10).

Clinically, scrub typhus presents as an acute febrile illness after an incubation period of 5 to 14 days. Most commonly fever, an eschar at the inoculation site, and lymphadenopathy occur, accompanied by headache, myalgia, and rash. The disease may vary from a mild, self-limiting infection to severe manifestations including multiorgan failure (11–13). Differences in the clinical course and manifestation of the disease have been observed, depending on the respective strain (14). Without treatment, the median case fatality rate is around 6%, but can rise to 70% in severe disease (15). Severe manifestations are driven by a hyperinflammatory host response. Once in the bloodstream, *O. tsutsugamushi* primarily infects endothelial cells and mononuclear phagocytes, triggering endothelial activation and disruption of vascular integrity while simultaneously promoting local cytokine release, which in turn only aggravates the proinflammatory state (16, 17).

In non-endemic regions, diagnosis is frequently delayed because of the non-specific presentation of the disease and the absence of awareness among clinicians. The eschar, a key diagnostic feature, is often overlooked or entirely absent, which is reflected in the widely heterogeneous reported prevalence, ranging from 40 to 92% (18, 19).

Importantly, conventional blood cultures and standard microbiological diagnostics are ineffective due to the intracellular nature of *Orientia tsutsugamushi*, making targeted molecular or serologic testing indispensable. As seroconversion typically occurs after 7 to 10 days after symptom onset, PCR remains the diagnostic method of choice in the early phase of infection. Eschar PCR offers higher sensitivity and a broader diagnostic window than whole-blood PCR, even after treatment initiation (20, 21).

Early consideration of scrub typhus as a differential diagnosis for fever in returning travelers from endemic areas is therefore critical to prevent diagnostic delay and allow timely initiation of specific therapy. Consequently, the clinical course of scrub typhus is typically benign and rapidly reversible.

## Case description

A 39-year-old woman presented to the emergency department with persistent high-grade fever up to 42 °C for 6 days, severe headache, nausea, and malaise. Additionally, the patient reported that her last bowel movement had occurred 5 days prior to admission, with two episodes of loose stool. At that time, she had returned from a 2-week vacation trip to Northern and Central Vietnam. She had not taken malaria prophylaxis and reported multiple insect bites during her stay in rural areas. Moreover, she denied contact with other animals. Her past medical history was unremarkable, and she was not taking regular medication.

At presentation, she appeared tired but alert and oriented. Her temperature was 37.8 °C, blood pressure 105/71 mmHg, and pulse 115 bpm. Oxygen saturation was 100% on ambient air. Cardiopulmonary auscultation was unremarkable, and no rash or petechiae were noted. Initially, no eschar was observed, only a small skin lesion under the armpit that was attributed to a mosquito bite. Abdominal examination revealed mild tenderness in the right upper quadrant.

**Figure 1:**
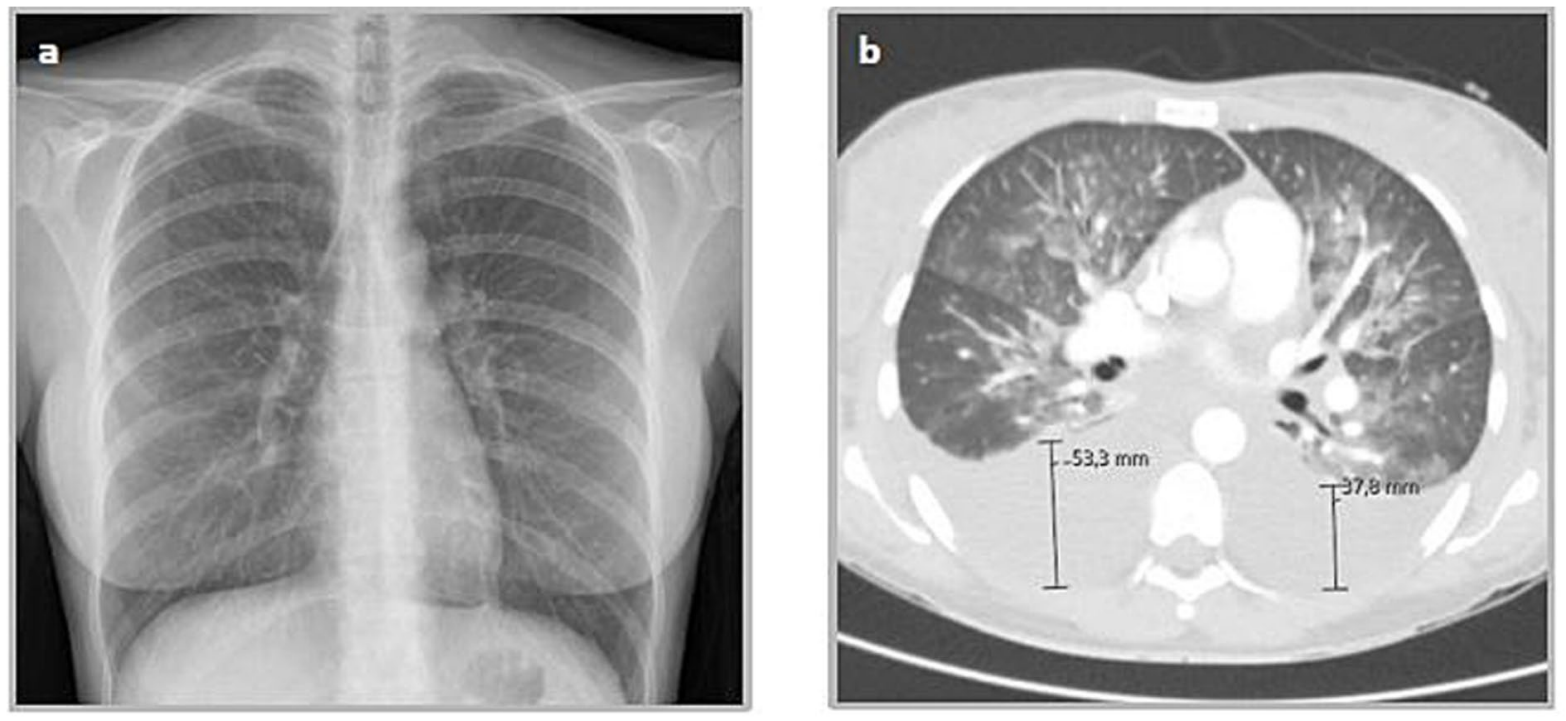
Illustration of the highly acute, dynamic course of the disease by radiological follow-up of the thorax after 3 days. **(a)** Unremarkable Chest X-ray in anterior-posterior-projection and standing position on the day of admission d0; **(b)** CT thorax images showing diffuse infiltrates and pleuraleffusions on d +3.

Initial laboratory results demonstrated normal leukocyte count (4.38 G/L), C-reactive protein was elevated (5.5 mg/dL), marked thrombocytopenia (95 G/L) and elevated LDH (717 U/L) with low haptoglobin (<10 mg/dL) indicating hemolysis. Hepatic enzymes were increased 8- to 10-fold the norm and bilirubin was slightly elevated, consistent with acute hepatocellular injury. The differential blood count also revealed eosinopenia without presence of atypical lymphocytes or reactive lymphocytosis. A chest X-ray was unremarkable (Figure 1a), while abdominal ultrasound revealed hepatosplenomegaly without biliary obstruction.

Besides standard testing for seasonal respiratory pathogens (*RSV, SARS-CoV-2, Influenza A/B*) and emergency testing for malaria (by using PCR, smear and rapid diagnostic test), a broad infectious work-up was initiated to cover the most common causes of fever in returning travellers from Vietnam: Common arboviral serologies (*dengue, chikungunya, Zika, West Nile virus*) showed no evidence of an acute infection and PCRs for *dengue, Zika* and *chikungunya virus* were negative, as were PCR and serology for *Leptospirosis* and screening for viral *hepatitis A, B, C* and *E* and *HIV*.

**Figure 2:**
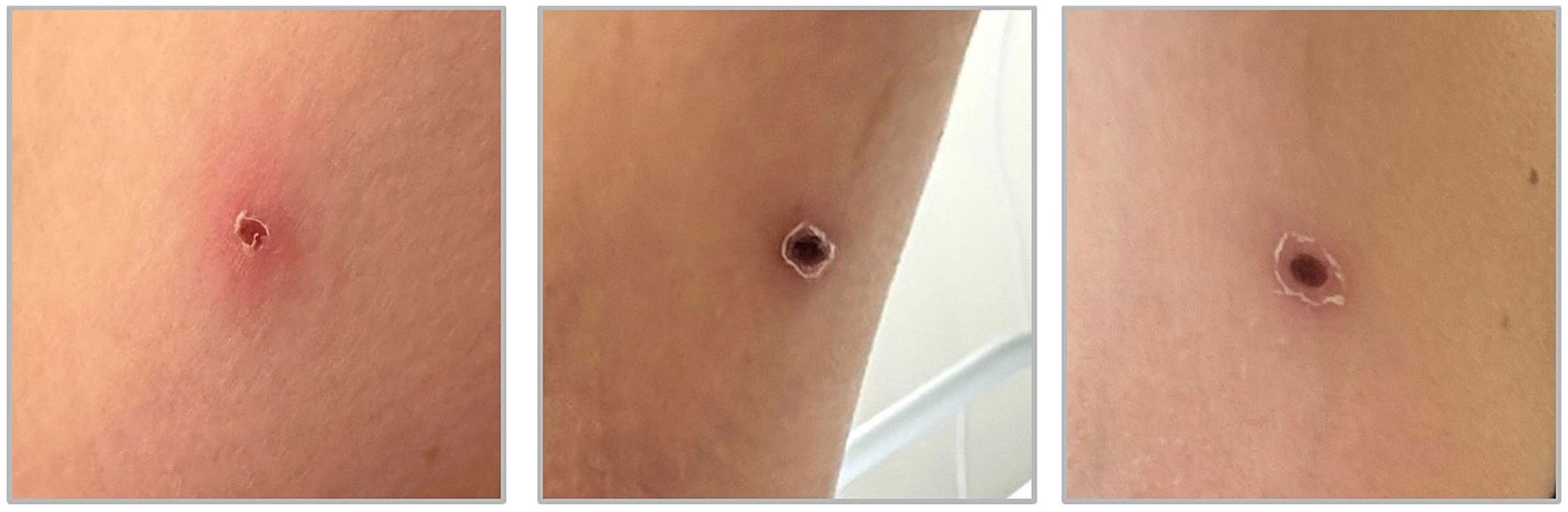
The images show the eschar of the patient in the left axilla, representing a bite-mark, probably of an *O. tsutsugamushi*-infected chigger, over the course of 1 week in relation to the day (d) of admission d0 (left to right: d-1; d + 5; d + 6) while symptom onset with fever occurred 6 days earlier on d-5.

Despite empirical treatment with ceftriaxone on the day of admission, the fever persisted, and the patient was transferred to the intensive care unit (ICU) the next day due to sepsis with a SOFA-Score of 4 (+2 platelets, +1 MAP <70 mmHg, +1 Bilirubin 1.6 mg/dL) as well as impending acute liver failure with rising transaminases (maximal AST 783 U/L, ALT 539 U/L) and signs of progressive hemolysis. Now, day seven after symptom onset, the lesion in the left axilla region, initially interpreted as an insect bite, increasingly resembled an eschar (Figure 2).

On day one and two after ICU admission, the patient developed increasing oxygen demand up to 4 L/min and presented with oxygen saturation drops to 80% at ambient air while presenting always alert and adequate. The CT-scan of the thorax on day 3 revealed bilateral ground-glass opacities, interlobular septal thickening, and moderate pleural effusions (right > left) with no evidence for pulmonary embolism, suggesting impending ARDS in the context of a systemic inflammatory response (Figure 1b). Abdominal CT confirmed periportal edema, splenomegaly, and minimal ascites, but no sign of abscess or cholestasis.

Echocardiography demonstrated normal biventricular function and no valvular abnormalities, excluding cardiac failure as a cause of the pleural effusions.

Given the travel history, epidemiology and clinical progression, doxycycline (100 mg orally twice a day) was added the day of admission to the ICU, respectively the second day of medical treatment (d+,1) to cover possible rickettsial infection, which was then considered as another differential diagnosis. Despite initial worsening, the patient became afebrile, oxygenation stabilized, and her general condition improved shortly after doxycycline was added. The coagulopathy showed sustained improvement under repeated administration of phytomenadione. Pleural effusions were managed with diuretics, and symptomatic measures were given for pain (headache, myalgia), fever, and nausea as needed. At this time point *Rickettsia* IgG antibodies (*R. conorii* and *R. typhi*) were not detected.

On day 7, a PCR from EDTA blood sample, which was sent to the Bernhard Nocht Institute in Hamburg, Germany (National Reference Center for Tropical Infectious Pathogens), returned positive for *Orientia tsutsugamushi*, confirming the diagnosis of scrub typhus. In line with an acute infection, IgM antibodies against *O. tsutsugamushi* tested positive (1,320, reference <1:40) by Indirect Immunofluorescence Test (IIFT), while IgG-antibodies mildly positive (1,160, reference <1:80).

**Figure 3:**
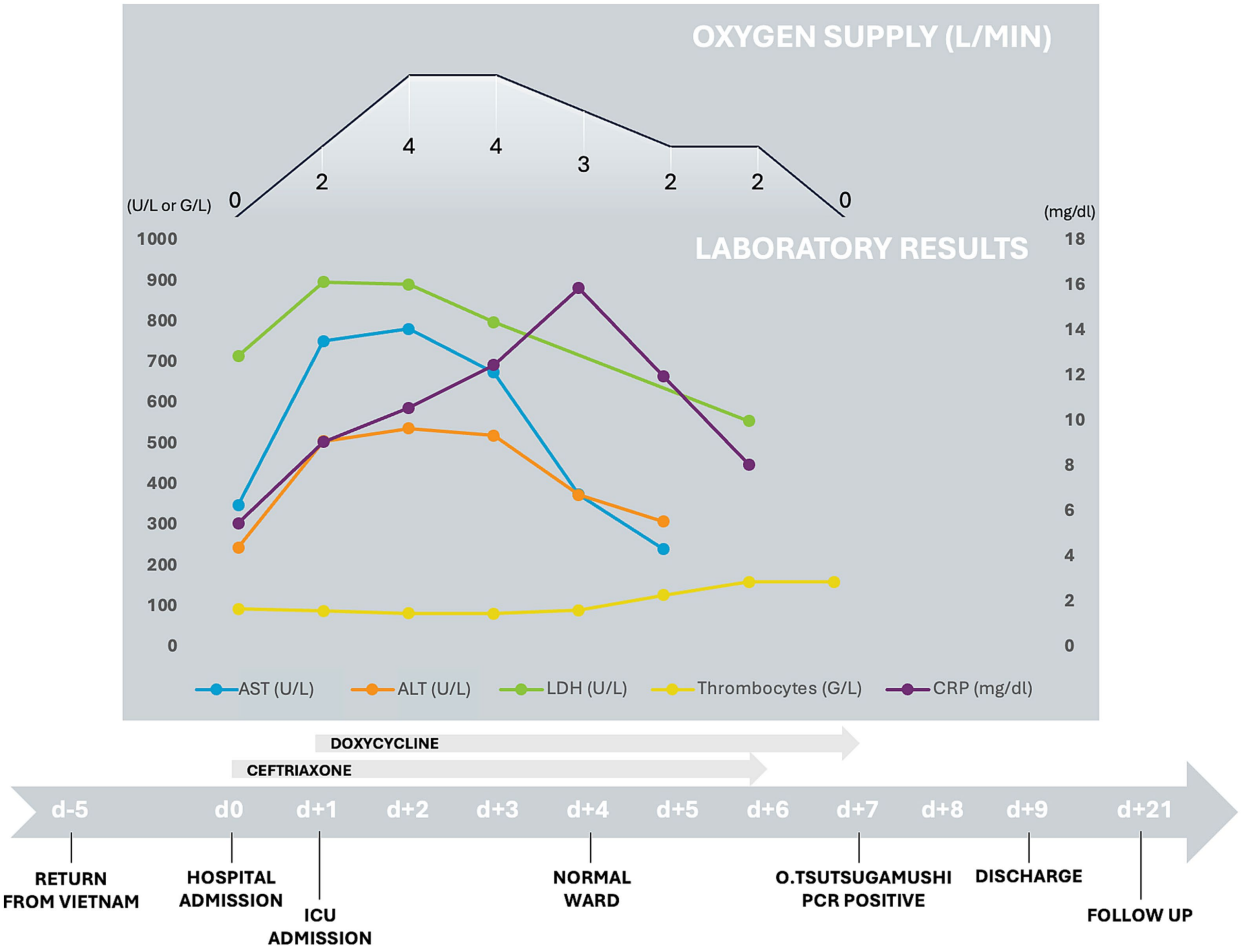
Timeline with course of relevant laboratory results, oxygen-supply and antimicrobial treatment. The symptom onset occurred on the day of return from Vietnam (d-5), 6 days later the first medical visit with hospital admission and start of ceftriaxone therapy (d0) and 15 days after symptom onset discharge (d + 9).

**Figure 4:**
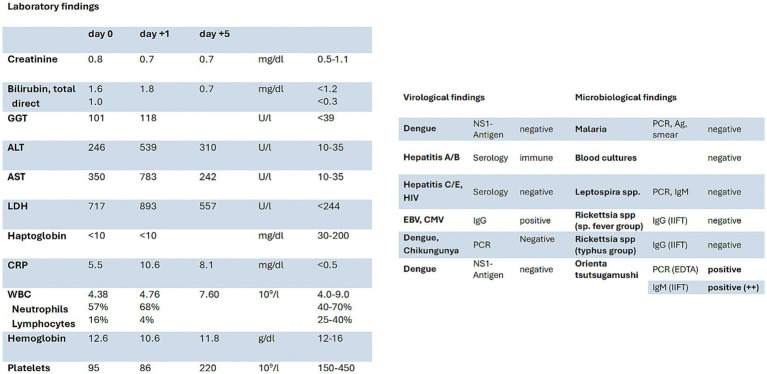
Summary of key laboratory parameters on day of admission (d0) with follow up on day +1 and d +5 (left), key microbiological and virological findings (right). Blood samples for *Leptospira, Ricksettia* spp. and *Orientia tsutsugamushi* were collected 8 days from fever onset.

To better understand the rapid clinical course of our patient a detailed timeline figure shows hallmarks of the disease as well as relevant laboratory findings and oxygen supply (Figure 3). Both laboratory and microbiological/virological findings can be seen in Figure 4.

Doxycycline therapy was continued for 7 days. Hepatic and inflammatory parameters, as well as markers of hemolysis gradually normalized, and thrombocyte counts steadily recovered. On day 7 upon admission, she no longer required oxygen supplementation. Follow-up sonography showed regression of pleural effusions under diuretic treatment. The patient was discharged after 9 days of hospitalization in good clinical condition and remained asymptomatic at 2-week telemedical follow-up.

## Discussion

Scrub fever is a neglected, but wide-spread and reemerging zoonotic infection. In Southeast Asia, *Orientia tsutsugamushi* is transmitted by larval trombiculid mites, particularly *Leptotrombidium species* inhabiting scrub vegetation and rural landscapes (10). Given the patient’s history, the most plausible exposure occurred during a rural hike and boat trip in central Vietnam following recent rainfall, conditions known to favor mite activity and human exposure.

The clinical manifestations of Scrub typhus range from mostly mild and asymptomatic courses to rare but severe multi-organ deterioration. Severe disease is driven by a combination of direct endothelial injury and indirect, macrophage-mediated hyperinflammation, leading to serious downstream consequences, including capillary leak, coagulopathy, thrombocytopenia and pulmonary involvement (16). The transition from acute infection to a hyperinflammatory state even with further progression to hemophagocytic lymphohistiocytosis (HLH) is well described and mostly halted by the timely initiation of targeted antimicrobials (22).

Generally, in travelers returning from Southeast Asia with febrile illness, thrombocytopenia, and elevated liver enzymes, several differential diagnoses must be considered. Dengue fever is among the most common causes of febrile thrombocytopenia in Vietnam and frequently presents with transaminitis and systemic inflammation (23). Malaria, particularly *Plasmodium falciparum*, must be rapidly excluded due to its potentially fulminant course (24). Leptospirosis should be considered in patients with freshwater exposure, as it is endemic in Vietnam and may present with fever, hepatitis, thrombocytopenia, and pulmonary manifestations (25). Enteric fever caused by *Salmonella Typhi* or *Paratyphi* may similarly present with sustained high fever, eosinopenia, and mild hepatitis (26). Other vector-borne intracellular bacterial infections may also present with similar clinical manifestations. Human granulocytic anaplasmosis HGA, a deer tick transmitted rickettsial infection caused by *Anaplasma phagocytophilum* has been reported to cause severe disease including pneumonia, acute respiratory distress syndrome, and septic shock, although cases in Southeast Asia appear to be rare (27). Finally, several spotted fever group rickettsioses occurring in Southeast Asia can also lead to severe systemic infection and pulmonary complications (28). Finally, scrub typhus caused by *Orientia tsutsugamushi* represents an important and often underrecognized cause of undifferentiated febrile illness in rural Southeast Asia, including Vietnam.

In this case of an *O. tsutsugamushi* infection progressing to sepsis in a traveler returning from Vietnam early specialist involvement, rapid consideration of differentials, and thus, timely initiation of adequate antiinfective therapy against rickettsial disease proved crucial. Although Berlin criteria for ARDS were not fully met, the patient demonstrated an early trajectory towards substantial respiratory deterioration with bilateral infiltrates, pleural effusions and rising inflammatory markers. This underscores the efficacy of timely targeted therapy – not only in preventing progression to septic shock, full-blown ARDS or Hemophagocytic lymphohistiocytosis, but also in ensuring full recovery without sequelae.

Hence, and due to emerging concerns regarding antimicrobial susceptibility, the appropriate anti-infective treatment regimen is of high interest. Early reports from Southeast Asia suggest reduced doxycycline responsiveness in certain *O. tsutsugamushi* strains, raising the possibility of developing resistance (29). While doxycycline and azithromycin remain the recommended first-line agents, the optimal management of severe scrub typhus is not fully defined. Intravenous doxycycline may be used where available. Rifampicin, although effective, should be reserved for situations in which active tuberculosis has been reliably excluded due to its potential to promote rifampicin-resistant *M. tuberculosis* (30). Lately, a randomized trial by Varghese et al. supported the consideration of combination therapy of doxycycline and azithromycine compared with monotherapy in severe disease via a composite of death from any cause at day 28, persistent complications at day 7, and persistent fever at day 5 with combination therapy. However, looking at 28-day mortality and adverse events alone no significant differences have been observed for either regime (31).

This case outlines the diagnostic challenge in non-endemic regions. Limited clinician familiarity leads to delayed recognition, while empiric sepsis regimens commonly rely on *β*-lactams not covering *O. tsutsugamushi* (32). Thorough physical examination – including deliberate inspection for an eschar - remains essential and might have hastened diagnosis by at least 1 day in the present case. Importantly, one must note that although the appearance of an eschar is pathognomonic, in many cases of Scrub typhus it does not occur (18).

In Germany, only 11 cases of imported scrub typhus have been diagnosed between 2010 and 2018, with pathogen detection by PCR in only two cases. Eight patients required hospitalization, and one patient developed severe disease with respiratory failure and meningoencephalitis. In this patient, an eschar was absent, and both diagnosis and doxycycline treatment were delayed. Septic features were not described in the case series, and detailed clinical courses were likewise not provided (16).

Given the rising incidence in endemic regions, expanding reported transmission zones, and increasing global mobility, scrub typhus must be considered in travelers with fever plus characteristic clinical and laboratory constellations with or without the presence of an eschar after returning from endemic areas. Accordingly, it should prompt specific testing for *O. tsutsugamushi* as well as adaptations of treatment.

## Data Availability

The original contributions presented in the study are included in the article/supplementary material, further inquiries can be directed to the corresponding author.
